# Genome Sequences of 12 Bacterial Isolates Obtained from the Urine of Pregnant Women

**DOI:** 10.1128/genomeA.00882-16

**Published:** 2016-09-29

**Authors:** Cory M. Weimer, Grace E. Deitzler, Lloyd S. Robinson, SoEun Park, Kymberlie Hallsworth-Pepin, Aye Wollam, Makedonka Mitreva, Warren G. Lewis, Amanda L. Lewis

**Affiliations:** a Department of Molecular Microbiology, Washington University School of Medicine, St. Louis, Missouri, USA; b Department of Obstetrics and Gynecology, Washington University School of Medicine, St. Louis, Missouri, USA; c Department of Medicine, Washington University School of Medicine, St. Louis, Missouri, USA; d Center for Women’s Infectious Disease Research, Washington University School of Medicine, St. Louis, Missouri, USA; e McDonnell Genome Institute, Washington University School of Medicine, St. Louis, Missouri, USA

## Abstract

The presence of bacteria in urine can pose significant risks during pregnancy. However, there are few reference genome strains for many common urinary bacteria. We isolated 12 urinary strains of *Streptococcus, Staphylococcus, Citrobacter, Gardnerella*, and *Lactobacillus*. These strains and their genomes are now available to the research community.

## GENOME ANNOUNCEMENT

Nearly 8 million urinary tract infections (UTIs) occur each year in the United States alone (1). During pregnancy, dilation of the renal pelvis and ureters makes women more prone to bacterial infection of the kidney. Landmark studies by Mittendorf and coworkers employed a randomized placebo-controlled trial showing that 40% of women with persistent (untreated) asymptomatic bacteriuria (ASB) went on to experience pyelonephritis, while none of the antibiotic-treated women went on experience this outcome (2). Due to the risks posed by pyelonephritis to pregnancy, it is now common practice in the United States to screen and treat pregnant women for asymptomatic bacteriuria.

*Escherichia coli* is the most common and best-studied cause of UTI. However, other less common bacteria are not well studied, despite being responsible for tens of thousands of cases in the United States alone. For example, group B *Streptococcus* (GBS) is a common vaginal bacterium responsible for ~1% of UTIs (1) among young sexually active women (~80,000 cases/year). GBS is also a leading cause of newborn sepsis (3) and commonly causes stillbirth (4) and placental infections in pregnant women (5). However, little is known about how GBS causes UTIs, and there are few urine isolates with sequenced genomes.

Similarly, there is an emerging understanding that the bladder is home to a surprising variety of bacteria, or at least that the bladder is frequently exposed to bacteria from nearby mucosal reservoirs (6). However, urinary strains from many taxa within these phyla are not readily available.

Here, we isolated and performed genome sequencing for 12 new strains of urinary bacteria. Briefly, clean-catch urine samples were provided by pregnant women as part of the Women and Infants’ Health Specimen Consortium (WIHSC) according to Washington University institutional review board (IRB)-approved protocol 20110382. Samples were sent from the Ob/Gyn clinic to the Barnes Jewish Hospital microbiology laboratory for identification of suspected uropathogens. Species identification was confirmed by full-length 16S rRNA gene sequence analysis. Genomic DNA was obtained using the Wizard purification kit (Promega). Genome assembly and annotation was performed as described more fully in the companion paper published in this issue (“Genome sequences of 11 human vaginal *Actinobacteria*”). Briefly, genomes were assembled *de novo* using the One Button Velvet assembly pipeline (version 1.1.06) (7). Gene products were predicted/annotated using GeneMark, Glimmer3 (8, 9), NCBI’s nonredundant bacterial (NR) database, and Pfam (10), tRNAscan-SE (11), RNAmmer (12), and Rfam (13).

### Accession number(s).

These whole-genome shotgun projects have been deposited in GenBank under the accession numbers listed in Table 1. The sequences described in this paper are the first versions. To facilitate future research in this field, we have also made the strains available to the research community by depositing them with the Biodefense and Emerging Infections (BEI) Research Resource Repository (see BEI numbers in Table 1).

**TABLE 1:** Strain identifiers and nucleotide accession numbers

Genus/species	Strain	BEI catalog no.	Nucleotide accession no.
*Streptococcus agalactiae*	PSS_7632A	HMS-1244	LRQM00000000
*Streptococcus agalactiae*	PSS_7632B	HMS-1245	LRQN00000000
*Streptococcus agalactiae*	PSS_7625	HMS-1246	LRQL00000000
*Streptococcus agalactiae*	PSS_7568	HMS-1247	LRQK00000000
*Streptococcus agalactiae*	PSS_7678	HMS-1248	LRQO00000000
*Streptococcus agalactiae*	PSS_7722	HMS-1249	LRQP00000000
*Streptococcus agalactiae*	PSS_7736	HMS-1250	LRQQ00000000
*Citrobacter koseri*	PSS_7778B	HMS-1275	LRPT00000000
*Gardnerella vaginalis*	PSS_7772B	HMS-1276	LRQB00000000
*Lactobacillus crispatus*	PSS7772C	HMS-1277	LSQY00000000
*Lactobacillus gasseri*	PSS7772D	HMS-1278	LRQD00000000
*Staphylococcus aureus*	PSS7673	HMS-1279	LRQH00000000
